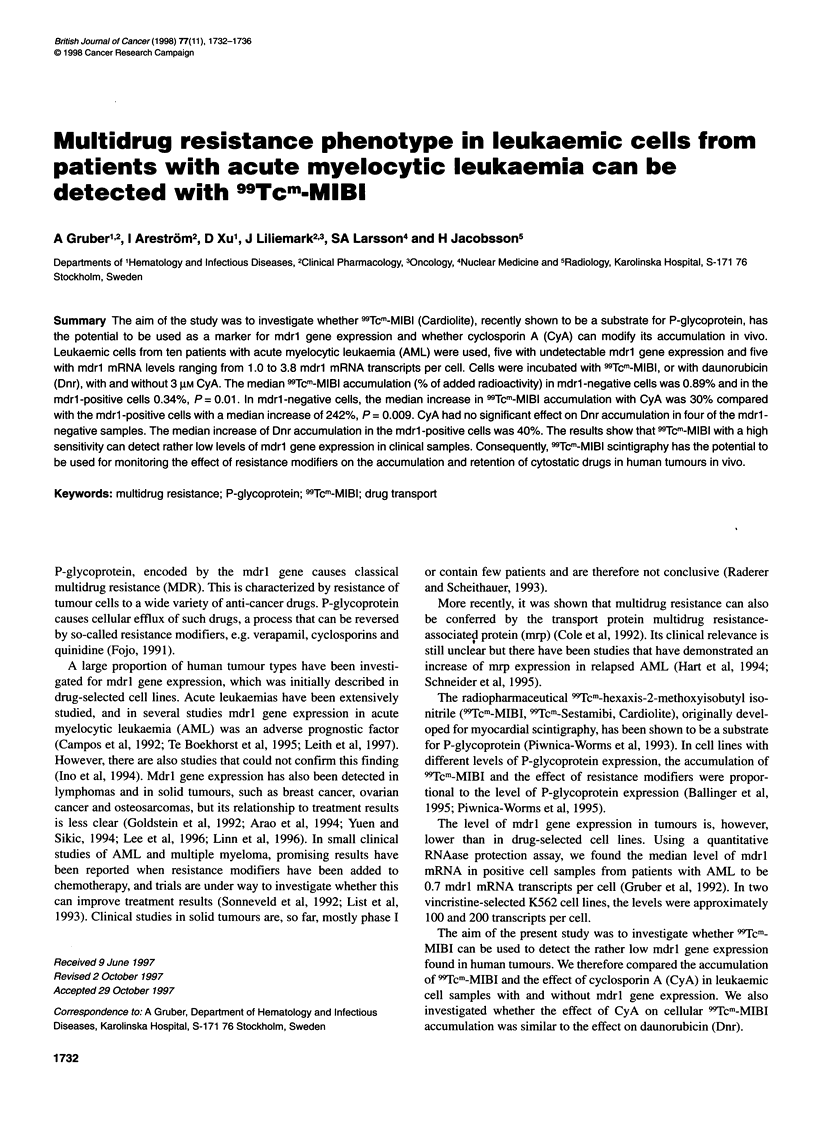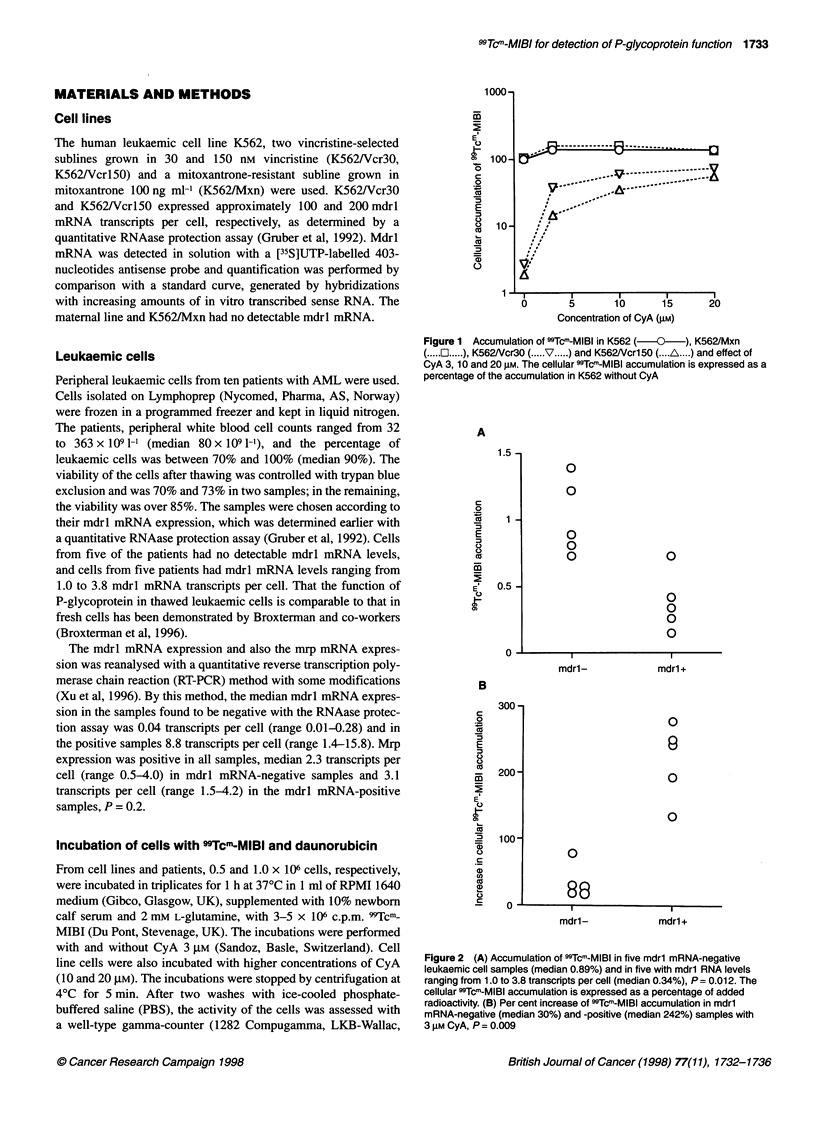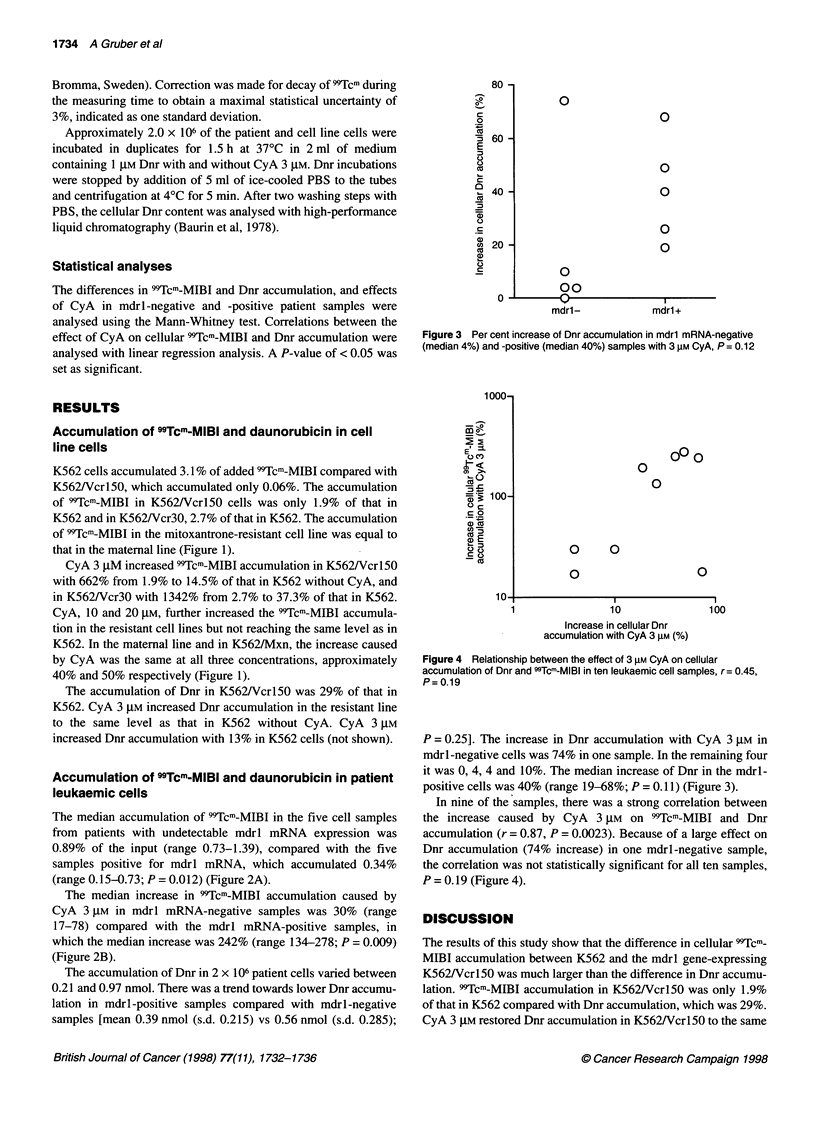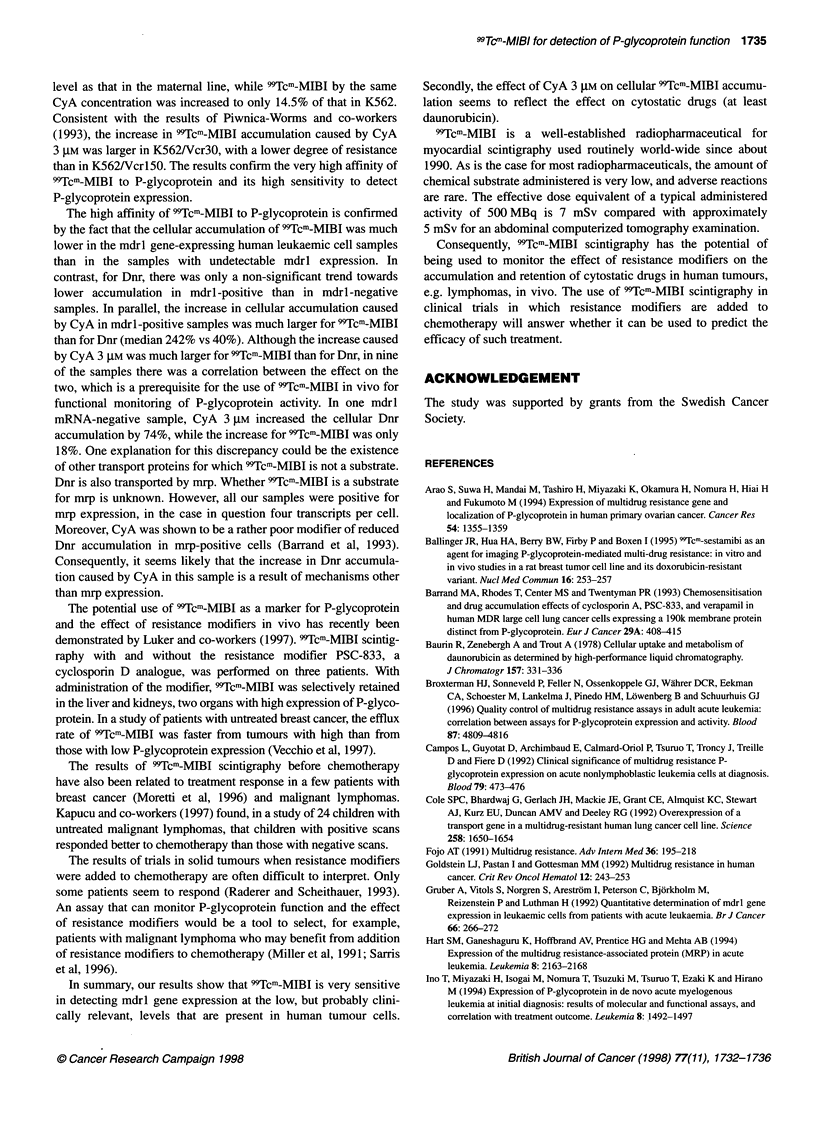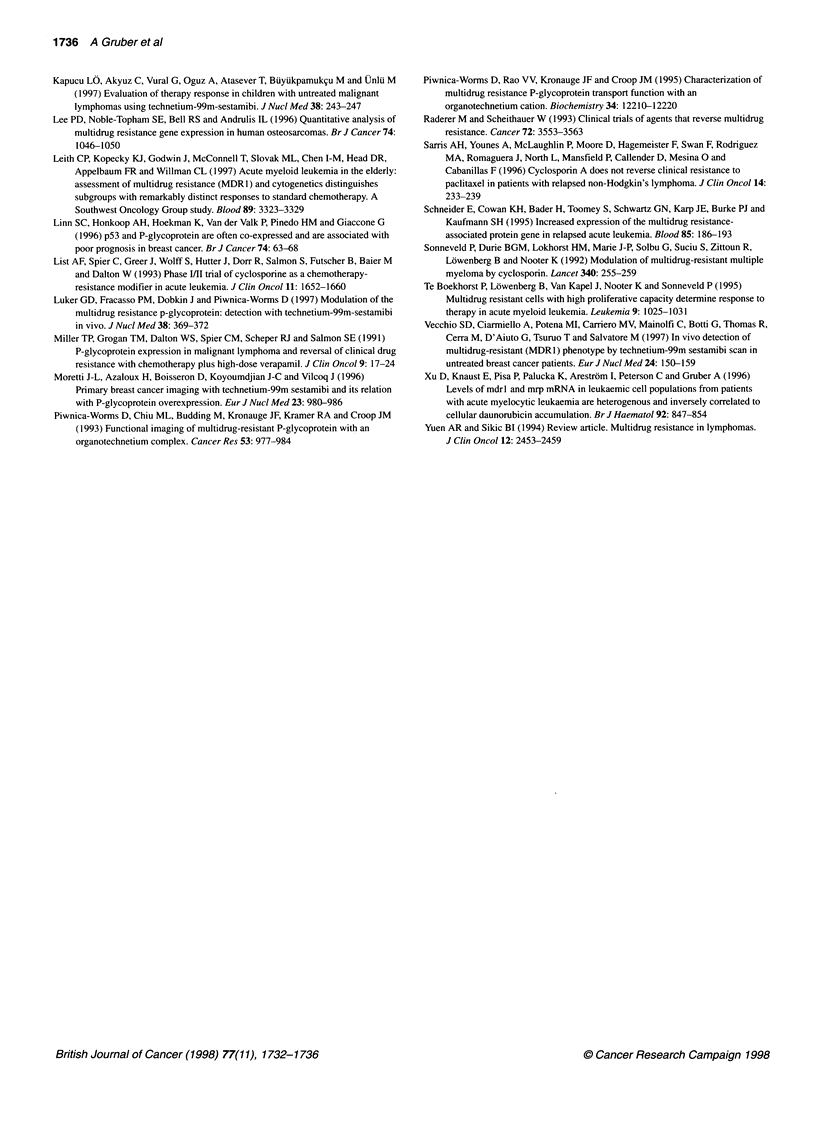# Multidrug resistance phenotype in leukaemic cells from patients with acute myelocytic leukaemia can be detected with 99Tc(m)-MIBI.

**DOI:** 10.1038/bjc.1998.290

**Published:** 1998-06

**Authors:** A. Gruber, I. ArestrÃ¶m, D. Xu, J. Liliemark, S. A. Larsson, H. Jacobsson

**Affiliations:** Department of Hematology and Infectious Diseases, Karolinska Hospital, Stockholm, Sweden.

## Abstract

The aim of the study was to investigate whether 99Tc(m)-MIBI (Cardiolite), recently shown to be a substrate for P-glycoprotein, has the potential to be used as a marker for mdr1 gene expression and whether cyclosporin A (CyA) can modify its accumulation in vivo. Leukaemic cells from ten patients with acute myelocytic leukaemia (AML) were used, five with undetectable mdr1 gene expression and five with mdr1 mRNA levels ranging from 1.0 to 3.8 mdr1 mRNA transcripts per cell. Cells were incubated with 99Tc(m)-MIBI, or with daunorubicin (Dnr), with and without 3 microM CyA. The median 99Tc(m)-MIBI accumulation (% of added radioactivity) in mdr1-negative cells was 0.89% and in the mdr1-positive cells 0.34%, P = 0.01. In mdr1-negative cells, the median increase in 99Tc(m)-MIBI accumulation with CyA was 30% compared with the mdr1-positive cells with a median increase of 242%, P = 0.009. CyA had no significant effect on Dnr accumulation in four of the mdr1-negative samples. The median increase of Dnr accumulation in the mdr1-positive cells was 40%. The results show that 99Tc(m)-MIBI with a high sensitivity can detect rather low levels of mdr1 gene expression in clinical samples. Consequently, 99T(c)m-MIBI scintigraphy has the potential to be used for monitoring the effect of resistance modifiers on the accumulation and retention of cytostatic drugs in human tumours in vivo.


British Journal of Cancer (1998) 77(11), 1732–1736
© 1998 Cancer Research Campaign

### Multidrug resistance phenotype in leukaemic cells from patients with acute myelocytic leukaemia can be detected with $^{99}$Tc$^m$-MIBI

**A Gruber[1,2], I Areström[2], D Xu[1], J Liliemark[2,3], SA Larsson[4] and H Jacobsson[5]**

Departments of [1]Hematology and Infectious Diseases, [2]Clinical Pharmacology, [3]Oncology, [4]Nuclear Medicine and [5]Radiology, Karolinska Hospital, S-171 76 Stockholm, Sweden

**Summary** The aim of the study was to investigate whether $^{99}$Tc$^m$-MIBI (Cardiolite), recently shown to be a substrate for P-glycoprotein, has the potential to be used as a marker for mdr1 gene expression and whether cyclosporin A (CyA) can modify its accumulation in vivo. Leukaemic cells from ten patients with acute myelocytic leukaemia (AML) were used, five with undetectable mdr1 gene expression and five with mdr1 mRNA levels ranging from 1.0 to 3.8 mdr1 mRNA transcripts per cell. Cells were incubated with $^{99}$Tc$^m$-MIBI, or with daunorubicin (Dnr), with and without 3 μM CyA. The median $^{99}$Tc$^m$-MIBI accumulation (% of added radioactivity) in mdr1-negative cells was 0.89% and in the mdr1-positive cells 0.34%, $P = 0.01$. In mdr1-negative cells, the median increase in $^{99}$Tc$^m$-MIBI accumulation with CyA was 30% compared with the mdr1-positive cells with a median increase of 242%, $P = 0.009$. CyA had no significant effect on Dnr accumulation in four of the mdr1-negative samples. The median increase of Dnr accumulation in the mdr1-positive cells was 40%. The results show that $^{99}$Tc$^m$-MIBI with a high sensitivity can detect rather low levels of mdr1 gene expression in clinical samples. Consequently, $^{99}$Tc$^m$-MIBI scintigraphy has the potential to be used for monitoring the effect of resistance modifiers on the accumulation and retention of cytostatic drugs in human tumours in vivo.

**Keywords:** multidrug resistance; P-glycoprotein; $^{99}$Tc$^m$-MIBI; drug transport

P-glycoprotein, encoded by the mdrl gene causes classical multidrug resistance (MDR). This is characterized by resistance of tumour cells to a wide variety of anti-cancer drugs. P-glycoprotein causes cellular efflux of such drugs, a process that can be reversed by so-called resistance modifiers, e.g. verapamil, cyclosporins and quinidine (Fojo, 1991).

A large proportion of human tumour types have been investigated for mdrl gene expression, which was initially described in drug-selected cell lines. Acute leukaemias have been extensively studied, and in several studies mdrl gene expression in acute myelocytic leukaemia (AML) was an adverse prognostic factor (Campos et al, 1992; Te Boekhorst et al, 1995; Leith et al, 1997). However, there are also studies that could not confirm this finding (Ino et al, 1994). Mdrl gene expression has also been detected in lymphomas and in solid tumours, such as breast cancer, ovarian cancer and osteosarcomas, but its relationship to treatment results is less clear (Goldstein et al, 1992; Arao et al, 1994; Yuen and Sikic, 1994; Lee et al, 1996; Linn et al, 1996). In small clinical studies of AML and multiple myeloma, promising results have been reported when resistance modifiers have been added to chemotherapy, and trials are under way to investigate whether this can improve treatment results (Sonneveld et al, 1992; List et al, 1993). Clinical studies in solid tumours are, so far, mostly phase I or contain few patients and are therefore not conclusive (Raderer and Scheithauer, 1993).

*Received 9 June 1997*
*Revised 2 October 1997*
*Accepted 29 October 1997*

*Correspondence to:* A Gruber, Department of Hematology and Infectious Diseases, Karolinska Hospital, S-171 76 Stockholm, Sweden

More recently, it was shown that multidrug resistance can also be conferred by the transport protein multidrug resistance-associated protein (mrp) (Cole et al, 1992). Its clinical relevance is still unclear but there have been studies that have demonstrated an increase of mrp expression in relapsed AML (Hart et al, 1994; Schneider et al, 1995).

The radiopharmaceutical $^{99}$Tc$^m$-hexaxis-2-methoxyisobutyl isonitrile ($^{99}$Tc$^m$-MIBI, $^{99}$Tc$^m$-Sestamibi, Cardiolite), originally developed for myocardial scintigraphy, has been shown to be a substrate for P-glycoprotein (Piwnica-Worms et al, 1993). In cell lines with different levels of P-glycoprotein expression, the accumulation of $^{99}$Tc$^m$-MIBI and the effect of resistance modifiers were proportional to the level of P-glycoprotein expression (Ballinger et al, 1995; Piwnica-Worms et al, 1995).

The level of mdrl gene expression in tumours is, however, lower than in drug-selected cell lines. Using a quantitative RNAase protection assay, we found the median level of mdrl mRNA in positive cell samples from patients with AML to be 0.7 mdrl mRNA transcripts per cell (Gruber et al, 1992). In two vincristine-selected K562 cell lines, the levels were approximately 100 and 200 transcripts per cell.

The aim of the present study was to investigate whether $^{99}$Tc$^m$-MIBI can be used to detect the rather low mdrl gene expression found in human tumours. We therefore compared the accumulation of $^{99}$Tc$^m$-MIBI and the effect of cyclosporin A (CyA) in leukaemic cell samples with and without mdrl gene expression. We also investigated whether the effect of CyA on cellular $^{99}$Tc$^m$-MIBI accumulation was similar to the effect on daunorubicin (Dnr).

### MATERIALS AND METHODS

### Cell lines

The human leukaemic cell line K562, two vincristine-selected sublines grown in 30 and 150 nM vincristine (K562/Vcr30, K562/Vcr150) and a mitoxantrone-resistant subline grown in mitoxantrone 100 ng $ml^{-1}$ (K562/Mxn) were used. K562/Vcr30 and K562/Vcr150 expressed approximately 100 and 200 mdrl mRNA transcripts per cell, respectively, as determined by a quantitative RNAase protection assay (Gruber et al, 1992). Mdrl mRNA was detected in solution with a $[^{35}S]$UTP-labelled 403-nucleotides antisense probe and quantification was performed by comparison with a standard curve, generated by hybridizations with increasing amounts of in vitro transcribed sense RNA. The maternal line and K562/Mxn had no detectable mdrl mRNA.

### Leukaemic cells

Peripheral leukaemic cells from ten patients with AML were used. Cells isolated on Lymphoprep (Nycomed, Pharma, AS, Norway) were frozen in a programmed freezer and kept in liquid nitrogen. The patients, peripheral white blood cell counts ranged from 32 to $363 \times 10^9\ l^{-1}$ (median $80 \times 10^9\ l^{-1}$), and the percentage of leukaemic cells was between 70% and 100% (median 90%). The viability of the cells after thawing was controlled with trypan blue exclusion and was 70% and 73% in two samples; in the remaining, the viability was over 85%. The samples were chosen according to their mdrl mRNA expression, which was determined earlier with a quantitative RNAase protection assay (Gruber et al, 1992). Cells from five of the patients had no detectable mdrl mRNA levels, and cells from five patients had mdrl mRNA levels ranging from 1.0 to 3.8 mdrl mRNA transcripts per cell. That the function of P-glycoprotein in thawed leukaemic cells is comparable to that in fresh cells has been demonstrated by Broxterman and co-workers (Broxterman et al, 1996).

The mdrl mRNA expression and also the mrp mRNA expression was reanalysed with a quantitative reverse transcription polymerase chain reaction (RT-PCR) method with some modifications (Xu et al, 1996). By this method, the median mdrl mRNA expression in the samples found to be negative with the RNAase protection assay was 0.04 transcripts per cell (range 0.01–0.28) and in the positive samples 8.8 transcripts per cell (range 1.4–15.8). Mrp expression was positive in all samples, median 2.3 transcripts per cell (range 0.5–4.0) in mdrl mRNA-negative samples and 3.1 transcripts per cell (range 1.5–4.2) in the mdrl mRNA-positive samples, $P = 0.2$.

### Incubation of cells with $^{99}Tc^m$-MIBI and daunorubicin

From cell lines and patients, 0.5 and $1.0 \times 10^6$ cells, respectively, were incubated in triplicates for 1 h at 37°C in 1 ml of RPMI 1640 medium (Gibco, Glasgow, UK), supplemented with 10% newborn calf serum and 2 mM L-glutamine, with $3–5 \times 10^6$ c.p.m. $^{99}Tc^m$-MIBI (Du Pont, Stevenage, UK). The incubations were performed with and without CyA 3 μM (Sandoz, Basle, Switzerland). Cell line cells were also incubated with higher concentrations of CyA (10 and 20 μM). The incubations were stopped by centrifugation at 4°C for 5 min. After two washes with ice-cooled phosphate-buffered saline (PBS), the activity of the cells was assessed with a well-type gamma-counter (1282 Compugamma, LKB-Wallac,

**Figure 1** Accumulation of $^{99}Tc^m$-MIBI in K562 (——O——), K562/Mxn (.....□.....), K562/Vcr30 (.....▽.....) and K562/Vcr150 (....△....) and effect of CyA 3, 10 and 20 μM. The cellular $^{99}Tc^m$-MIBI accumulation is expressed as a percentage of the accumulation in K562 without CyA

**Figure 2** (**A**) Accumulation of $^{99}Tc^m$-MIBI in five mdr1 mRNA-negative leukaemic cell samples (median 0.89%) and in five with mdr1 RNA levels ranging from 1.0 to 3.8 transcripts per cell (median 0.34%), $P = 0.012$. The cellular $^{99}Tc^m$-MIBI accumulation is expressed as a percentage of added radioactivity. (**B**) Per cent increase of $^{99}Tc^m$-MIBI accumulation in mdr1 mRNA-negative (median 30%) and -positive (median 242%) samples with 3 μM CyA, $P = 0.009$

© Cancer Research Campaign 1998

Bromma, Sweden). Correction was made for decay of $^{99}Tc^m$ during the measuring time to obtain a maximal statistical uncertainty of 3%, indicated as one standard deviation.

Approximately $2.0 \times 10^6$ of the patient and cell line cells were incubated in duplicates for 1.5 h at 37°C in 2 ml of medium containing 1 μM Dnr with and without CyA 3 μM. Dnr incubations were stopped by addition of 5 ml of ice-cooled PBS to the tubes and centrifugation at 4°C for 5 min. After two washing steps with PBS, the cellular Dnr content was analysed with high-performance liquid chromatography (Baurin et al, 1978).

### Statistical analyses

The differences in $^{99}Tc^m$-MIBI and Dnr accumulation, and effects of CyA in mdrl-negative and -positive patient samples were analysed using the Mann-Whitney test. Correlations between the effect of CyA on cellular $^{99}Tc^m$-MIBI and Dnr accumulation were analysed with linear regression analysis. A $P$-value of $< 0.05$ was set as significant.

### RESULTS

### Accumulation of $^{99}Tc^m$-MIBI and daunorubicin in cell line cells

K562 cells accumulated 3.1% of added $^{99}Tc^m$-MIBI compared with K562/Vcr150, which accumulated only 0.06%. The accumulation of $^{99}Tc^m$-MIBI in K562/Vcr150 cells was only 1.9% of that in K562 and in K562/Vcr30, 2.7% of that in K562. The accumulation of $^{99}Tc^m$-MIBI in the mitoxantrone-resistant cell line was equal to that in the maternal line (Figure 1).

CyA 3 μM increased $^{99}Tc^m$-MIBI accumulation in K562/Vcr150 with 662% from 1.9% to 14.5% of that in K562 without CyA, and in K562/Vcr30 with 1342% from 2.7% to 37.3% of that in K562. CyA, 10 and 20 μM, further increased the $^{99}Tc^m$-MIBI accumulation in the resistant cell lines but not reaching the same level as in K562. In the maternal line and in K562/Mxn, the increase caused by CyA was the same at all three concentrations, approximately 40% and 50% respectively (Figure 1).

The accumulation of Dnr in K562/Vcr150 was 29% of that in K562. CyA 3 μM increased Dnr accumulation in the resistant line to the same level as that in K562 without CyA. CyA 3 μM increased Dnr accumulation with 13% in K562 cells (not shown).

### Accumulation of $^{99}Tc^m$-MIBI and daunorubicin in patient leukaemic cells

The median accumulation of $^{99}Tc^m$-MIBI in the five cell samples from patients with undetectable mdrl mRNA expression was 0.89% of the input (range 0.73–1.39), compared with the five samples positive for mdrl mRNA, which accumulated 0.34% (range 0.15–0.73; $P = 0.012$) (Figure 2A).

The median increase in $^{99}Tc^m$-MIBI accumulation caused by CyA 3 μM in mdrl mRNA-negative samples was 30% (range 17–78) compared with the mdrl mRNA-positive samples, in which the median increase was 242% (range 134–278; $P = 0.009$) (Figure 2B).

The accumulation of Dnr in $2 \times 10^6$ patient cells varied between 0.21 and 0.97 nmol. There was a trend towards lower Dnr accumulation in mdrl-positive samples compared with mdrl-negative samples [mean 0.39 nmol (s.d. 0.215) vs 0.56 nmol (s.d. 0.285); $P = 0.25$]. The increase in Dnr accumulation with CyA 3 μM in mdrl-negative cells was 74% in one sample. In the remaining four it was 0, 4, 4 and 10%. The median increase of Dnr in the mdrl-positive cells was 40% (range 19–68%; $P = 0.11$) (Figure 3).

**Figure 3** Per cent increase of Dnr accumulation in mdr1 mRNA-negative (median 4%) and -positive (median 40%) samples with 3 μM CyA, $P = 0.12$

**Figure 4** Relationship between the effect of 3 μM CyA on cellular accumulation of Dnr and $^{99}Tc^m$-MIBI in ten leukaemic cell samples, $r = 0.45$, $P = 0.19$

In nine of the samples, there was a strong correlation between the increase caused by CyA 3 μM on $^{99}Tc^m$-MIBI and Dnr accumulation ($r = 0.87$, $P = 0.0023$). Because of a large effect on Dnr accumulation (74% increase) in one mdrl-negative sample, the correlation was not statistically significant for all ten samples, $P = 0.19$ (Figure 4).

### DISCUSSION

The results of this study show that the difference in cellular $^{99}Tc^m$-MIBI accumulation between K562 and the mdrl gene-expressing K562/Vcr150 was much larger than the difference in Dnr accumulation. $^{99}Tc^m$-MIBI accumulation in K562/Vcr150 was only 1.9% of that in K562 compared with Dnr accumulation, which was 29%. CyA 3 μM restored Dnr accumulation in K562/Vcr150 to the same

© Cancer Research Campaign 1998

level as that in the maternal line, while $^{99}Tc^{m}$-MIBI by the same CyA concentration was increased to only 14.5% of that in K562. Consistent with the results of Piwnica-Worms and co-workers (1993), the increase in $^{99}Tc^{m}$-MIBI accumulation caused by CyA 3 µM was larger in K562/Vcr30, with a lower degree of resistance than in K562/Vcr150. The results confirm the very high affinity of $^{99}Tc^{m}$-MIBI to P-glycoprotein and its high sensitivity to detect P-glycoprotein expression.

The high affinity of $^{99}Tc^{m}$-MIBI to P-glycoprotein is confirmed by the fact that the cellular accumulation of $^{99}Tc^{m}$-MIBI was much lower in the mdr1 gene-expressing human leukaemic cell samples than in the samples with undetectable mdr1 expression. In contrast, for Dnr, there was only a non-significant trend towards lower accumulation in mdr1-positive than in mdr1-negative samples. In parallel, the increase in cellular accumulation caused by CyA in mdr1-positive samples was much larger for $^{99}Tc^{m}$-MIBI than for Dnr (median 242% vs 40%). Although the increase caused by CyA 3 µM was much larger for $^{99}Tc^{m}$-MIBI than for Dnr, in nine of the samples there was a correlation between the effect on the two, which is a prerequisite for the use of $^{99}Tc^{m}$-MIBI in vivo for functional monitoring of P-glycoprotein activity. In one mdr1 mRNA-negative sample, CyA 3 µM increased the cellular Dnr accumulation by 74%, while the increase for $^{99}Tc^{m}$-MIBI was only 18%. One explanation for this discrepancy could be the existence of other transport proteins for which $^{99}Tc^{m}$-MIBI is not a substrate. Dnr is also transported by mrp. Whether $^{99}Tc^{m}$-MIBI is a substrate for mrp is unknown. However, all our samples were positive for mrp expression, in the case in question four transcripts per cell. Moreover, CyA was shown to be a rather poor modifier of reduced Dnr accumulation in mrp-positive cells (Barrand et al, 1993). Consequently, it seems likely that the increase in Dnr accumulation caused by CyA in this sample is a result of mechanisms other than mrp expression.

The potential use of $^{99}Tc^{m}$-MIBI as a marker for P-glycoprotein and the effect of resistance modifiers in vivo has recently been demonstrated by Luker and co-workers (1997). $^{99}Tc^{m}$-MIBI scintigraphy with and without the resistance modifier PSC-833, a cyclosporin D analogue, was performed on three patients. With administration of the modifier, $^{99}Tc^{m}$-MIBI was selectively retained in the liver and kidneys, two organs with high expression of P-glycoprotein. In a study of patients with untreated breast cancer, the efflux rate of $^{99}Tc^{m}$-MIBI was faster from tumours with high than from those with low P-glycoprotein expression (Vecchio et al, 1997).

The results of $^{99}Tc^{m}$-MIBI scintigraphy before chemotherapy have also been related to treatment response in a few patients with breast cancer (Moretti et al, 1996) and malignant lymphomas. Kapucu and co-workers (1997) found, in a study of 24 children with untreated malignant lymphomas, that children with positive scans responded better to chemotherapy than those with negative scans.

The results of trials in solid tumours when resistance modifiers were added to chemotherapy are often difficult to interpret. Only some patients seem to respond (Raderer and Scheithauer, 1993). An assay that can monitor P-glycoprotein function and the effect of resistance modifiers would be a tool to select, for example, patients with malignant lymphoma who may benefit from addition of resistance modifiers to chemotherapy (Miller et al, 1991; Sarris et al, 1996).

In summary, our results show that $^{99}Tc^{m}$-MIBI is very sensitive in detecting mdr1 gene expression at the low, but probably clinically relevant, levels that are present in human tumour cells. Secondly, the effect of CyA 3 µM on cellular $^{99}Tc^{m}$-MIBI accumulation seems to reflect the effect on cytostatic drugs (at least daunorubicin).

$^{99}Tc^{m}$-MIBI is a well-established radiopharmaceutical for myocardial scintigraphy used routinely world-wide since about 1990. As is the case for most radiopharmaceuticals, the amount of chemical substrate administered is very low, and adverse reactions are rare. The effective dose equivalent of a typical administered activity of 500 MBq is 7 mSv compared with approximately 5 mSv for an abdominal computerized tomography examination.

Consequently, $^{99}Tc^{m}$-MIBI scintigraphy has the potential of being used to monitor the effect of resistance modifiers on the accumulation and retention of cytostatic drugs in human tumours, e.g. lymphomas, in vivo. The use of $^{99}Tc^{m}$-MIBI scintigraphy in clinical trials in which resistance modifiers are added to chemotherapy will answer whether it can be used to predict the efficacy of such treatment.

### ACKNOWLEDGEMENT

The study was supported by grants from the Swedish Cancer Society.

### REFERENCES

Arao S, Suwa H, Mandai M, Tashiro H, Miyazaki K, Okamura H, Nomura H, Hiai H and Fukumoto M (1994) Expression of multidrug resistance gene and localization of P-glycoprotein in human primary ovarian cancer. *Cancer Res* **54**: 1355–1359

Ballinger JR, Hua HA, Berry BW, Firby P and Boxen I (1995) $^{99}Tc^{m}$-sestamibi as an agent for imaging P-glycoprotein-mediated multi-drug resistance: in vitro and in vivo studies in a rat breast tumor cell line and its doxorubicin-resistant variant. *Nucl Med Commun* **16**: 253–257

Barrand MA, Rhodes T, Center MS and Twentyman PR (1993) Chemosensitisation and drug accumulation effects of cyclosporin A, PSC-833, and verapamil in human MDR large cell lung cancer cells expressing a 190k membrane protein distinct from P-glycoprotein. *Eur J Cancer* **29A**: 408–415

Baurin R, Zenebergh A and Trout A (1978) Cellular uptake and metabolism of daunorubicin as determined by high-performance liquid chromatography. *J Chromatogr* **157**: 331–336

Broxterman HJ, Sonneveld P, Feller N, Ossenkoppele GJ, Wáhrer DCR, Eekman CA, Schoester M, Lankelma J, Pinedo HM, Löwenberg B and Schuurhuis GJ (1996) Quality control of multidrug resistance assays in adult acute leukemia: correlation between assays for P-glycoprotein expression and activity. *Blood* **87**: 4809–4816

Campos L, Guyotat D, Archimbaud E, Calmard-Oriol P, Tsuruo T, Troncy J, Treille D and Fiere D (1992) Clinical significance of multidrug resistance P-glycoprotein expression on acute nonlymphoblastic leukemia cells at diagnosis. *Blood* **79**: 473–476

Cole SPC, Bhardwaj G, Gerlach JH, Mackie JE, Grant CE, Almquist KC, Stewart AJ, Kurz EU, Duncan AMV and Deeley RG (1992) Overexpression of a transport gene in a multidrug-resistant human lung cancer cell line. *Science* **258**: 1650–1654

Fojo AT (1991) Multidrug resistance. *Adv Intern Med* **36**: 195–218

Goldstein LJ, Pastan I and Gottesman MM (1992) Multidrug resistance in human cancer. *Crit Rev Oncol Hematol* **12**: 243–253

Gruber A, Vitols S, Norgren S, Areström I, Peterson C, Björkholm M, Reizenstein P and Luthman H (1992) Quantitative determination of mdr1 gene expression in leukaemic cells from patients with acute leukaemia. *Br J Cancer* **66**: 266–272

Hart SM, Ganeshaguru K, Hoffbrand AV, Prentice HG and Mehta AB (1994) Expression of the multidrug resistance-associated protein (MRP) in acute leukemia. *Leukemia* **8**: 2163–2168

Ino T, Miyazaki H, Isogai M, Nomura T, Tsuzuki M, Tsuruo T, Ezaki K and Hirano M (1994) Expression of P-glycoprotein in de novo acute myelogenous leukemia at initial diagnosis: results of molecular and functional assays, and correlation with treatment outcome. *Leukemia* **8**: 1492–1497

© Cancer Research Campaign 1998 

Kapucu LÖ, Akyuz C, Vural G, Oguz A, Atasever T, Büyükpamukçu M and Ünlü M (1997) Evaluation of therapy response in children with untreated malignant lymphomas using technetium-99m-sestamibi. *J Nucl Med* **38**: 243–247

Lee PD, Noble-Topham SE, Bell RS and Andrulis IL (1996) Quantitative analysis of multidrug resistance gene expression in human osteosarcomas. *Br J Cancer* **74**: 1046–1050

Leith CP, Kopecky KJ, Godwin J, McConnell T, Slovak ML, Chen I-M, Head DR, Appelbaum FR and Willman CL (1997) Acute myeloid leukemia in the elderly: assessment of multidrug resistance (MDR1) and cytogenetics distinguishes subgroups with remarkably distinct responses to standard chemotherapy. A Southwest Oncology Group study. *Blood* **89**: 3323–3329

Linn SC, Honkoop AH, Hoekman K, Van der Valk P, Pinedo HM and Giaccone G (1996) p53 and P-glycoprotein are often co-expressed and are associated with poor prognosis in breast cancer. *Br J Cancer* **74**: 63–68

List AF, Spier C, Greer J, Wolff S, Hutter J, Dorr R, Salmon S, Futscher B, Baier M and Dalton W (1993) Phase I/II trial of cyclosporine as a chemotherapy-resistance modifier in acute leukemia. *J Clin Oncol* **11**: 1652–1660

Luker GD, Fracasso PM, Dobkin J and Piwnica-Worms D (1997) Modulation of the multidrug resistance p-glycoprotein: detection with technetium-99m-sestamibi in vivo. *J Nucl Med* **38**: 369–372

Miller TP, Grogan TM, Dalton WS, Spier CM, Scheper RJ and Salmon SE (1991) P-glycoprotein expression in malignant lymphoma and reversal of clinical drug resistance with chemotherapy plus high-dose verapamil. *J Clin Oncol* **9**: 17–24

Moretti J-L, Azaloux H, Boisseron D, Koyoumdjian J-C and Vilcoq J (1996) Primary breast cancer imaging with technetium-99m sestamibi and its relation with P-glycoprotein overexpression. *Eur J Nucl Med* **23**: 980–986

Piwnica-Worms D, Chiu ML, Budding M, Kronauge JF, Kramer RA and Croop JM (1993) Functional imaging of multidrug-resistant P-glycoprotein with an organotechnetium complex. *Cancer Res* **53**: 977–984

Piwnica-Worms D, Rao VV, Kronauge JF and Croop JM (1995) Characterization of multidrug resistance P-glycoprotein transport function with an organotechnetium cation. *Biochemistry* **34**: 12210–12220

Raderer M and Scheithauer W (1993) Clinical trials of agents that reverse multidrug resistance. *Cancer* **72**: 3553–3563

Sarris AH, Younes A, McLaughlin P, Moore D, Hagemeister F, Swan F, Rodriguez MA, Romaguera J, North L, Mansfield P, Callender D, Mesina O and Cabanillas F (1996) Cyclosporin A does not reverse clinical resistance to paclitaxel in patients with relapsed non-Hodgkin's lymphoma. *J Clin Oncol* **14**: 233–239

Schneider E, Cowan KH, Bader H, Toomey S, Schwartz GN, Karp JE, Burke PJ and Kaufmann SH (1995) Increased expression of the multidrug resistance-associated protein gene in relapsed acute leukemia. *Blood* **85**: 186–193

Sonneveld P, Durie BGM, Lokhorst HM, Marie J-P, Solbu G, Suciu S, Zittoun R, Löwenberg B and Nooter K (1992) Modulation of multidrug-resistant multiple myeloma by cyclosporin. *Lancet* **340**: 255–259

Te Boekhorst P, Löwenberg B, Van Kapel J, Nooter K and Sonneveld P (1995) Multidrug resistant cells with high proliferative capacity determine response to therapy in acute myeloid leukemia. *Leukemia* **9**: 1025–1031

Vecchio SD, Ciarmiello A, Potena MI, Carriero MV, Mainolfi C, Botti G, Thomas R, Cerra M, D'Aiuto G, Tsuruo T and Salvatore M (1997) In vivo detection of multidrug-resistant (MDR1) phenotype by technetium-99m sestamibi scan in untreated breast cancer patients. *Eur J Nucl Med* **24**: 150–159

Xu D, Knaust E, Pisa P, Palucka K, Areström I, Peterson C and Gruber A (1996) Levels of mdr1 and mrp mRNA in leukaemic cell populations from patients with acute myelocytic leukaemia are heterogenous and inversely correlated to cellular daunorubicin accumulation. *Br J Haematol* **92**: 847–854

Yuen AR and Sikic BI (1994) Review article. Multidrug resistance in lymphomas. *J Clin Oncol* **12**: 2453–2459

© Cancer Research Campaign 1998